# Maximising the clustering coefficient of networks and the effects on habitat network robustness

**DOI:** 10.1371/journal.pone.0240940

**Published:** 2020-10-20

**Authors:** Henriette Heer, Lucas Streib, Ralf B. Schäfer, Stefan Ruzika

**Affiliations:** 1 Department of Quantitative Landscape Ecology, iES Landau, University of Koblenz-Landau, Landau i.d. Pfalz, Germany; 2 Department of Mathematics, University of Kaiserslautern, Kaiserslautern, Germany; Universidad Rey Juan Carlos, SPAIN

## Abstract

The robustness of networks against node failure and the response of networks to node removal has been studied extensively for networks such as transportation networks, power grids, and food webs. In many cases, a network’s clustering coefficient was identified as a good indicator for network robustness. In ecology, habitat networks constitute a powerful tool to represent metapopulations or -communities, where nodes represent habitat patches and links indicate how these are connected. Current climate and land-use changes result in decline of habitat area and its connectivity and are thus the main drivers for the ongoing biodiversity loss. Conservation efforts are therefore needed to improve the connectivity and mitigate effects of habitat loss. Habitat loss can easily be modelled with the help of habitat networks and the question arises how to modify networks to obtain higher robustness. Here, we develop tools to identify which links should be added to a network to increase the robustness. We introduce two different heuristics, Greedy and Lazy Greedy, to maximize the clustering coefficient if multiple links can be added. We test these approaches and compare the results to the optimal solution for different generic networks including a variety of standard networks as well as spatially explicit landscape based habitat networks. In a last step, we simulate the robustness of habitat networks before and after adding multiple links and investigate the increase in robustness depending on both the number of added links and the heuristic used. We found that using our heuristics to add links to sparse networks such as habitat networks has a greater impact on the clustering coefficient compared to randomly adding links. The Greedy algorithm delivered optimal results in almost all cases when adding two links to the network. Furthermore, the robustness of networks increased with the number of additional links added using the Greedy or Lazy Greedy algorithm.

## Introduction

Habitat loss and fragmentation due to changes in climate and land use are one of the main drivers of the ongoing global biodiversity crisis [1–5]. The loss and fragmentation of habitat lead to a decrease in habitat connectivity, impeding the movement of individuals between patches [6, 7]. This dispersal is crucial for species survival, as it facilitates interaction such as the exchange of genes between different populations and thus allows for the existence of metapopulations—a “population of populations” [8–10]. As a consequence of the constantly intensifying climate and land-use change, it is important for species conservation that we particularly try to preserve and improve habitat connectivity by creating dispersal corridors increasing a landscape’s permeability [11–14].

Graph theory provides powerful tools to represent and analyse habitat connectivity in highly fragmented landscapes [15–18]. Here, metapopulations are represented by habitat networks where nodes represent habitat patches and links indicate how these are connected [16, 19, 20]. With the help of habitat networks, the loss of habitat can easily be represented by removing nodes and reduced connectivity by removing links from the network [21–23]. Accordingly, many studies apply graph-theoretic tools to evaluate the effect of climate and land-use change and to find solutions for these effects in landscape planning [24–26].

The resilience of networks against node and link removal, also called network robustness, has been studied in a variety of networks, such as transportation networks, power grids, and food webs [27–31]. A network’s clustering coefficient was identified as a good proxy for robustness in a variety of networks such as habitat networks of herbivores and brown bears [32–34]. The clustering coefficient of a network was proposed by Watts and Strogatz [35] and is defined as the average of the local clustering coefficient of its nodes. A node’s clustering coefficient measures how close its neighbourhood is to a complete network in terms of the relative density of links in its neighbourhood. We exploit the relationship between the clustering coefficient and network robustness and improve a network’s robustness by maximising the network’s clustering coefficient.

The question we pose in this work is: Where should additional links best be created within a habitat network to maximise its clustering coefficient? We propose an algorithm to identify the missing link of a network that leads to the biggest increase in network robustness when added to the network, by using the clustering coefficient as an indicator. We introduce two different heuristics, a Greedy algorithm [36] and a deducted Lazy Greedy algorithm, to maximize the clustering coefficient if multiple links can be added. To speed up the two algorithms, we developed a method to update the clustering coefficient of a network after adding one link as opposed to calculating it without any prior knowledge. Both approaches can be applied to any network, regardless of whether or not it is based on a spatial component. We test these approaches and compare the results to the optimal solution for different generic networks including a variety of standard networks independent of space as well as spatially explicit landscape based habitat networks.

In a last step, we simulate the robustness of habitat networks against habitat loss as proposed by Heer et al. [51] before and after adding multiple links and investigate the increase in robustness depending on both the number of added links and the used heuristic. The robustness simulation combines the simulation of habitat loss by randomly removing habitat patches from the network with the simulations of metapopulation dynamics to evaluate the metapopulation’s robustness. Our proposed methods thus provide tools to facilitate landscape restoration by identifying which location leads to the largest improvement when additional links are added in these places.

## Methods

### Outline of analysis

We present the algorithm to update the clustering coefficient after one link is added and the Greedy and Lazy Greedy algorithms to add more than one link. We evaluated the effect of adding links using the proposed algorithms on the clustering coefficient and therefore on the habitat network’s robustness. To evaluate the effect of the proposed algorithms on the clustering coefficient, we added two links to a variety of networks using (1) the Greedy algorithm, (2) the Lazy Greedy algorithm, and (3) a purely random approach. The clustering coefficients of the resulting networks were then compared to the clustering coefficient of the original network as well as the optimal solution, which was found by complete enumeration, i.e. iterating over all pairs of potential links. We tested our algorithms on different network types, including sparse standard networks (random, small-world, and regular) [37], dense standard networks, and habitat networks based on artificial landscapes and a generic insect species with both terrestrial and aquatic life stages created by Streib et al. [38].

Finally, we evaluated the effect of modified habitat networks on metapopulation robustness. To this end, we simulated and evaluated the metapopulation robustness as presented by Heer et al. [51] and studied the increase in robustness after adding links using the Greedy algorithm, the Lazy Greedy algorithm, and a random insertion approach. For these simulations, only the landscape-based habitat networks were taken into account as the standard networks are in general poor representatives of habitat networks.

### Notation

We use the following notation throughout the manuscript. Let *G* = (*V*, *E*) be a simple, undirected, loopless network with node set *V* and link set *E* ⊂ *V* × *V*.

Let (*u*, *v*) ∈ *V* × *V* \ *E* be a pair of unconnected nodes in *G*. To be able to compare the network *G* with the extended network that arises from *G* by adding the link (*u*, *v*) to *G*, we use the following notation and set *G*′ ≔ (*V*, *E* ∪ {(*u*, *v*)}). If we want to emphasize the link (*u*, *v*), we will write *G* + *uv* ≔ *G*′.

**Fig 1:**
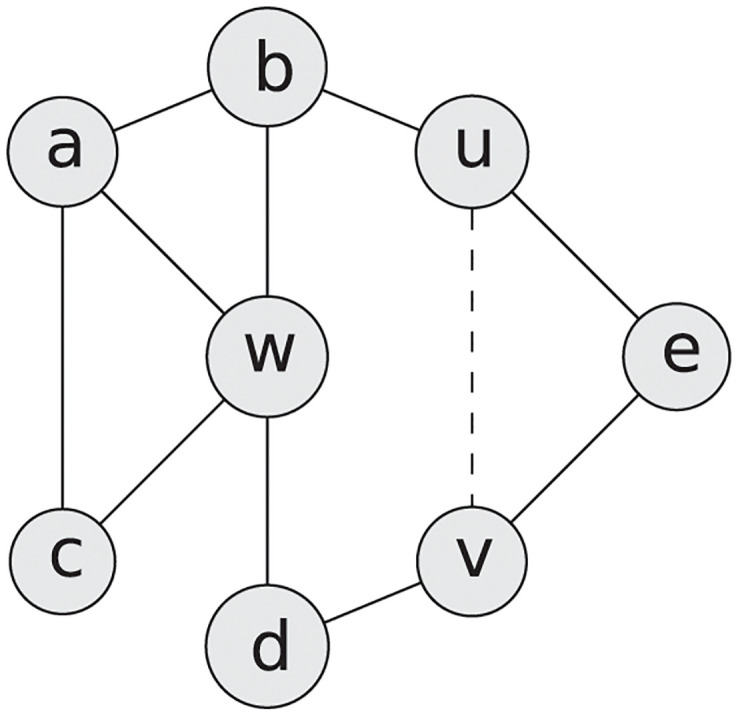
Example network to illustrate notation. *G* = (*V*, *E*) with *n* = 8 nodes, 10 links, *V* = {*a*, *b*, *c*, *d*, *e*, *u*, *v*, *w*}, and *E* = {(*a*, *b*), (*a*, *w*), (*a*, *c*), (*b*, *u*), (*b*, *w*), (*u*, *e*), (*w*, *c*), (*w*, *d*), (*e*, *v*), (*d*, *v*)}. We choose *m* = 1 link from the set E=V×V\E of all links not included in *G*. *G*′ is the network *G* after link (*u*, *v*) (represented as dashed line) was added: *G*′ ≔ *G* + *uv*. Then *N*(*w*) = {*a*, *b*, *c*, *d*}, $d_{w}=d_{w}^{\prime }=4$, *T*(*w*) = *T*′(*w*) = 2 (the triangles *abw* and *acw*) and $C\left(w\right)=\frac{2\cdot 2}{4(4-1)}=\frac{1}{3}$. For *u* we obtain *d*_*u*_ = 2 and $d_{u}^{\prime }=3$ and similarly *T*(*u*) = 0 and *T*′(*u*) = 1 (the triangle *uev*). *N*(*u*, *v*) = {*e*} and *k* = |*N*(*u*, *v*)| = 1. The clustering coefficient of *G* equals $C_{G}=\frac{1}{8}\cdot \left(\frac{1}{3}+0+0+\frac{1}{3}+\frac{2}{3}+1+0+0\right)=\frac{1}{8}\cdot \frac{7}{3}=\frac{7}{24}$ and the clustering coefficient of the extended network is $C_{G\prime }=\frac{1}{8}\cdot \left(\frac{1}{3}+\frac{1}{3}+\frac{1}{3}+\frac{1}{3}+\frac{2}{3}+1+0+1\right)=\frac{1}{8}\cdot 4=\frac{1}{2}$.

For a node *w* ∈ *V*, we set *N*(*w*) ≔ {*v* ∈ *V* : (*w*, *v*) ∈ *E*} as the set of neighbours of *w*, *d*_*w*_ ≔ |*N*(*w*)| as the degree, i.e. the number of neighbours, of *w* in *G* and $d_{w}^{\prime }$ the degree of *w* in *G*′. A triangle in a network *G* is a clique of three nodes {*u*, *v*, *w*}, i.e. all three nodes are connected with each other by links: (*u*, *v*), (*u*, *w*), (*v*, *w*) ∈ *E*. We set *T*(*w*) ≔ |{(*u*, *v*) ∈ *E* : *u*, *v* ∈ *N*(*w*)}| as the number of triangles in *G* that involve *w* and *T*′(*w*) as the number of triangles in *G*′. Furthermore, with *N*(*u*, *v*) ≔ *N*(*u*) ∩ *N*(*v*) we denote the set of common neighbours of *u* and *v* and *k* ≔ |*N*(*u*, *v*)| the number of common neighbours (Fig 1).

The clustering coefficient of a node *v* ∈ *V* is defined as

$$\begin{array}{c} C\left(v\right)=\{\begin{array}{cc} \frac{2T(v)}{d_{v}\left(d_{v}-1\right)} & \;\text{if}\;d_{v}>1\\ 0 & \;\text{if}\;d_{v}⩽1 \end{array}. \end{array}$$

It measures how close its neighbourhood is to a complete network in terms of the relative density of links in its neighbourhood. If all links between neighbours of *v* are present, then $T\left(v\right)=\frac{1}{2}d_{v}\left(d_{v}-1\right)$ and the clustering coefficient takes its maximum value of 1. If no links between neighbours are present, then *T*(*v*) = 0 and thus *C*(*v*) = 0.

The clustering coefficient of a network *G* with *n* ≔ |*V*| nodes is defined as the average over the clustering coefficient of its nodes:

$$\begin{array}{c} C_{G}=\frac{1}{n}\sum_{v\in V}C\left(v\right) \end{array}$$

and can take any value between 0 and 1. Computing the clustering coefficient of a network with *n* ≔ |*V*| nodes has an $O(n^{\omega })$ complexity with *ω* ⩽ 2.376 [39]. The most complex part of computing the clustering coefficient is finding triangles in a network, which can be done in $O(n^{\omega })$ using the adjacency matrix and fast matrix multiplication. Let m∈N be the number of links we want to add to the network and E⊆V×V\E a set of missing links to choose these *m* links from. Fig 1 gives an example for each variable introduced here.

Our aim is to improve a network’s robustness by adding links to the network. As the clustering coefficient is a good proxy for robustness [23, 32–34], we want to identify those links that should be added to the network to maximize the clustering coefficient. Mathematically, we want to solve the following problem:

**Problem 1**. *Let* *G* = (*V*, *E*) *be a network as above*, E⊆V×V\E, *and* *m* ⩾ 1 *be given*. *Find a subset* $\{e_{1},\ldots ,e_{m}\}\subseteq E$ *such that* *G*′ ≔ (*V*, *E* ∪ {*e*_1_, …, *e*_*m*_}) *has maximum clustering coefficient*. *In other words*, *find a solution to*

$$\begin{array}{cc} \max & \;C_{G\prime }\\ s.t.\; & G\prime =\left(V,E\cup \left\{e_{1},\ldots ,e_{m}\right\}\right)\\ & \{e_{1},\ldots ,e_{m}\}\subseteq E. \end{array}$$

**Example 1**. *Consider the network* *G* = (*V*, *E*) *from* Fig 1. *We set* E=V×V\E *and* *m* = 1, *i.e*. *we allow all unconnected pairs of nodes to be connected and the task is to identify* *m* = 1 *pair that maximizes the clustering coefficient when connecting the pair and adding the link to* *G*. *Problem 1 has two solutions*, *the pair* (*u*, *v*) *as well as the pair* (*d*, *e*), *which both increase the clustering coefficient to 0.5*. *If we set* *m* = 2 *in the same problem*, *we obtain the unique solution* (*b*, *e*) *and* (*d*, *e*) *with a new clustering coefficient of 0.625*.

In some cases, we want to add any link to the network in order to maximize the clustering coefficient and it makes sense to find those potential links {*e*_1_, …, *e*_*m*_} within all pairs of unconnected nodes. In this case we set E≔V×V\E. However, especially when considering habitat networks, we may want to restrict this set to only some pairs of unconnected nodes. For habitat networks, for example, we may want to restrict the set to those pairs of unconnected nodes that are within a certain (Euclidean) distance from each other. This represents the assumption that the species in focus has a limited dispersal distance independent of the underlying land-use class [40].

### Update clustering coefficient

We first aim to solve Problem 1 for *m* = 1, i.e., we want to find the pair of nodes (u,v)∈E, such that the network *G*′ = *G* + *uv* has maximum clustering coefficient.

A naïve approach to find the relevant nodes *u* and *v* is to iterate over all unconnected pairs of nodes, connect those, and calculate the clustering coefficient of the extended network from scratch. This has a run time of $O(|E|n^{\omega })$, as we iterate over |E| pairs and calculate the clustering coefficient each time from scratch. To speed up the process, however, we can exploit the fact that adding the link does not affect the clustering coefficient in most nodes. To see this, consider the degree of each node in *G* as well as the number of triangles it is part of. The degrees of the nodes in *G*′ equal the degrees of the nodes in *G*, except for the two nodes *u* and *v*, as adding (*u*, *v*) to *G* increases the degrees of *u* and *v* by exactly one. The number of triangles in *u* and *v* each increases by the number of common neighbours of *u* and *v*, as each common neighbour *w* ∈ *N*(*u*, *v*) introduces the triangle *uvw* and every triangle that does not use the link (*u*, *v*) also exists in *G*. Similarly, the number of triangles for each common neighbour of *u* and *v* increases by exactly one. The number of triangles does not change for every other node that is not *u*, *v* or a common neighbor of *u* and *v*. Accordingly, we can calculate the clustering coefficient of *G*′ by adding the difference caused by *u*, *v* and every common neighbour *w* of *u* and *v* to the original clustering coefficient *C*_*G*_:

$$\begin{array}{c} C_{G\prime }=C_{G}+\frac{1}{n}(\Delta C\left(u\right)+\Delta C\left(v\right)+\sum_{w\in N(u,v)}\frac{2}{d_{w}\left(d_{w}-1\right)}) \end{array}$$ (1)

with

$$\begin{array}{c} \Delta C\left(u\right)=\{\begin{array}{cc} \frac{2k\left(d_{u}-1\right)-4T\left(u\right)}{d_{u}\left(d_{u}^{2}-1\right)} & \;\text{if}\;d_{u}>1\\ 1 & \;\text{if}\;d_{u}=1 \end{array}. \end{array}$$

See S1 File for the proof of Eq 1.

**Fig 2:**
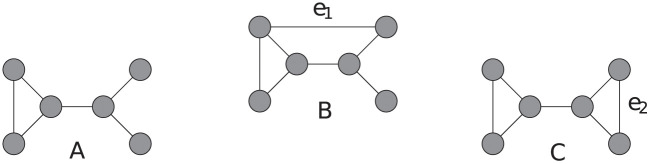
Varying effects of adding a link to a network on the clustering coefficient. (a) Original network with clustering coefficient $C_{G}=0.3\bar{8}$. (b) Network after connecting two nodes with no common neighbours, $C_{G+e_{1}}=0.2\bar{7}$. (c) Network after connecting two nodes with a common neighbour, $C_{G+e_{2}}=0.\bar{7}$.

It follows from Eq 1 and Fig 2, that adding a link to a network may also result in a smaller clustering coefficient compared to the original network. If *u* and *v* have no common neighbours, the sum over all common neighbours in Eq 1 is empty (and thus equals 0) and

$$\Delta C\left(u\right)=\frac{2\cdot 0\cdot \left(d_{u}-1\right)-4T\left(u\right)}{d_{u}\left(d_{u}^{2}-1\right)}=\frac{-4T(u)}{d_{u}\left(d_{u}^{2}-1\right)}⩽0.$$

Similarly, Δ*C*(*v*) ⩽ 0 and

$$C_{G}^{\prime }=C_{G}+\frac{1}{n}(\Delta C(u)+\Delta C(v)+0)⩽C_{G}.$$

Using Eq 1, we can update the clustering coefficient after adding a new link (*u*, *v*) to a network *G* = (*V*, *E*) with known clustering coefficient *C*.

**Algorithm 1** Update clustering coefficient

1: **procedure** UpdateClustering(*G* = (*V*, *E*), *C*_*G*_, (*u*, *v*))

2:  *C*_max_ ← 0

3:  *T* ← Triangles(*G*)

4:  *CN* ← CommonNeighbours(*u*, *v*)

5:  *k* = |*CN*|

6:  **if** *d*_*u*_ > 1 **then**

7:   $C_{\max}\leftarrow C_{\max}+\frac{2k\left(d_{u}-1\right)-4T\left(u\right)}{d_{u}\left(d_{u}^{2}-1\right)}$

8:  **else** *C*_max_ ← *C*_max_ + 1

9:  **if** *d*_*v*_ > 1 **then**

10:   $C_{\max}\leftarrow C_{\max}+\frac{2k\left(d_{v}-1\right)-4T\left(v\right)}{d_{v}\left(d_{v}^{2}-1\right)}$

11:  **else** *C*_max_ ← *C*_max_ + 1

12:  **for** *w* ∈ CN **do**

13:   $C_{\max}\leftarrow C_{\max}+\frac{2}{d_{w}\left(d_{w}-1\right)}$

14:  $C_{\max}\leftarrow \frac{C_{\max}}{\left|V\right|}$

15:  *C*_max_ ← *C*_max_ + *C*_*G*_

16:  **return** *C*_max_

Algorithm 1 takes a network *G* = (*V*, *E*), its clustering coefficient *C*, and a pair of unconnected nodes *u* and *v* as input and returns the clustering coefficient of the extended network *G* + *uv* using Eq 1. It finds the set of common neighbours of *u* and *v*, calculates Δ*C*(*u*) and Δ*C*(*v*), and then iterates over the set of common neighbours of *u* and *v* and increases the sum of Δ*C*(*u*) and Δ*C*(*v*) by $\frac{2}{d_{w}\left(d_{w}-1\right)}$ for each common neighbour *w*. The result is then averaged over the number of nodes in *G* and added to the original clustering coefficient. Eq 1 proves the correctness of this algorithm.

We use Algorithm 1 to develop a faster algorithm than the naïve one to find a solution of Problem 1 for *m* = 1. It iterates over the set E of all possible pairs of nodes and calculates the new clustering coefficient by updating the clustering coefficient of the original network.

**Algorithm 2** Maximize clustering coefficient

1: **procedure** MaximizeClustering(*G* = (*V*, *E*), E)

2:  *C*_*G*_ ← Clustering(*G*)

3:  *T* ← Triangles(*G*)

4:  *C*_max_ ← *C*_*G*_

5:  **for** (u,v)∈E **do**

6:   *C* = UpdateClustering(*G*, *C*_*G*_, (*u*, *v*))

7:   **if** *C* > *C*_max_ **then**

8:    *C*_max_ ← *C*

9:    *e*_max_ ← (*u*, *v*)

10:  **return** *C*_max_, *e*_max_

Algorithm 2 iterates over all potential links, uses Algorithm 1 to update the clustering coefficient and returns a link that yields the maximum clustering coefficient. As Algorithm 1 with input (*u*, *v*) returns the clustering coefficient of the extended network *G* + *uv*, and Algorithm 2 iterates over all potential links, it returns an optimal solution of Problem 1 for *m* = 1 in $O(n^{\omega }+|E|d_{\max})$, where *d*_max_ is the maximum degree of the nodes in *V*.

The algorithm solves Problem 1 reasonably fast for *m* = 1. When adding multiple links, however, every combination of potential links needs to be checked, slowing the procedure substantially down even for only two links: There are (|E|m) combinations of potential links and executing Algorithm 2 for each combination has a complexity of O(nω+(|E|m)|E|dmax). We thus introduce two heuristics, Greedy and Lazy Greedy, that identify the maximum clustering coefficient of a network when multiple links can be added.

### Greedy

The Greedy algorithm successively adds one link that maximizes the clustering coefficient of the current network. Starting with a network *G*, the algorithm iterates over the set E of all possible pairs of nodes and connects the pair *u*, *v* with the biggest increase in the clustering coefficient (see Algorithm 2). It then iterates again over all possible pairs of nodes in *G*′ to find the second link and continues, until *m* links were found.

**Algorithm 3** Greedy

1: **procedure** Greedy(*G* = (*V*, *E*), E, *m*)

2:  **for** *i* ∈ [1, *m*] **do**

3:   *C*, *e*_*i*_ = MaximizeClustering(*G* = (*V*, *E*), E)

4:   *G* = *G* + *e*

5:  **return** *e*_1_, …, *e*_*m*_

**Fig 3:**
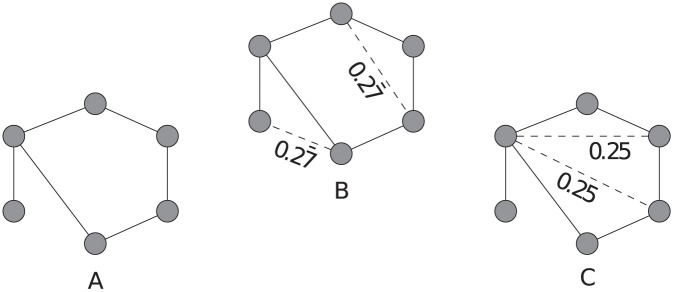
Example of non-optimal behaviour of the Greedy algorithm. (a) Original network with clustering coefficient *C* = 0. (b) Network with two links selected using the Greedy algorithm and clustering coefficient $C=0.\bar{5}$. (c) Optimal solution with clustering coefficient $C=0.60\bar{5}$. The value corresponding to the dashed lines show the increase of clustering coefficient by adding the corresponding link to the network in (a). After adding one of the links depicted as dashed lines to the network in (c), the contribution of the other link increases to $0.3\bar{5}$, as the two nodes incident to that link now have one common neighbour more (see Eq 1).

We can calculate the clustering coefficient and the number of triangles once and then update these numbers. In that case, the Greedy algorithm calculates the solution in $O(n^{\omega }+|E|d_{\max}m)$, as it executes Algorithm 2 exactly *m* times. However, the solution found by the Greedy algorithm 3 is not necessarily optimal: Consider the network depicted in Fig 3a and assume we can add two links. The Greedy algorithm will add the links shown in Fig 3b, while the links shown in Fig 3c lead to a higher clustering coefficient.

#### Lazy Greedy

For even faster calcuations—at the cost of optimality—we introduce a second heuristic, that iterates over all potential links once and then picks the *m* links that have the highest increase in the clustering coefficient if they were to be added individually.

The Lazy Greedy algorithm executes Algorithm 2 once and sorts the results afterwards. Using quick sort, sorting can be done in O(|E|log(|E|)) and we obtain a run time of $O(n^{\omega }+\left|E\right|d_{\max}+\left|E|\log(|E|\right)$ [41, 42]. Similar to the Greedy algorithm, the Lazy Greedy algorithm does not necessarily find the optimal solution to Problem 1. Fig 3 also serves as example of a non-optimal solution, as the Lazy Greedy algorithm will select the same links as the Greedy algorithm.

**Algorithm 4** Lazy Greedy

1: **procedure** LazyGreedy(*G* = (*V*, *E*), E, *m*)

2:  *C*_*G*_ ← Clustering(*G*)

3:  *T* ← Triangles(*G*)

4:  results ← new Array

5:  **for** (u,v)∈E **do**

6:   *C* = UpdateClustering(*G*, *C*_*G*_, (*u*, *v*))

7:   append (*u*, *v*, *C*) to results

8:  results ← sort results by *C*

9:  **return** results[1], results[m]

### Random approach

We compared the described heuristics to the results of a random approach, where links were added uniformly at random to a network.

### Networks

**Fig 4:**
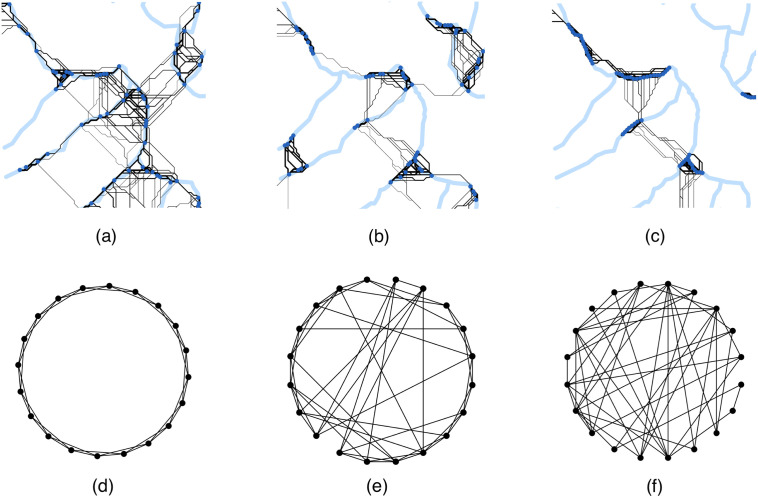
Networks examined. (a)–(c): Landscape-based networks. Dark-blue dots indicate nodes (habitat patches), black lines indicate links (dispersal pathways). The light-blue lines indicate the underlying stream network structure. (a) random allocation of habitat patches, (b) clustered allocation, (c) linear allocation. (d)-(f): Standard networks. (d) regular network, (e) small-world network, (f) random network.

We tested the described heuristics on a variety of network types, namely landscape-based habitat networks created by Streib et al. [38] with random, clustered, and contiguous allocation of habitat patches / nodes as well as networks common in mathematics (random, regular, and small-world networks) [37]. The random, regular, and small-world networks represent a variety of network structures and are widely used in many disciplines, such as engineering, social sciences, finance, biology, and also ecology [35, 37, 43–46]. Fig 4 shows examples of the networks.

#### Landscape-based habitat networks

The landscape-based habitat networks were set up by Streib et al. [38] based on a generic insect species with aquatic and terrestrial life stages, landscapes consisting of different landscape types associated with varying dispersal cost, and a 50 km × 50 km section of a real stream network from southwest Germany. The stream network section was divided into 25 tiles of 10 km × 10 km areas and intersected with an artificial landscape consisting of open agricultural land, forestry land, and urban area with associated dispersal costs. A subset containing 10% of the pixels in the real stream network were chosen as habitat patches. We considered 3 types of habitat patch arrangements leading to 3 types of landscape-based habitat networks, namely (1) random (with all habitat patches randomly selected along streams with equal probability), (2) clustered (with only some habitat patches randomly selected along streams with equal probability and the others randomly selected along streams with equal probability within a given radius around any of the initially selected habitat patches), and (3) contiguous (with a smaller fraction of habitat patches randomly selected along streams with equal probability and a larger fraction of others randomly selected along streams with equal probability within a given radius around any of the initially selected habitat patches, leading to a more contiguous arrangement of the habitat patches compared to the clustered allocation). Reflecting the different stream structures in the different landscape tiles, this results in habitat networks with 54 to 111 habitat patches. Habitat patches were connected with the help of a least-cost path analysis based on the dispersal cost in the underlying landscape. If the cummulative dispersal cost between two habitat patches was less than the maximum dispersal cost, the two patches were considered to be connected and a corresponding link was added to the network. Differing from Streib et al. [38], we assumed shorter dispersal ranges of about 1300m through open agricultural land to simulate particular sensitive species. These dispersal ranges translated to maximum dispersal costs of 650 (as we assumed a cost of 50 to traverse a 25m × 25m area of open agricultural land, see [38] for further information). To ensure that all network types have similar distributions of the number of links, we finally adjusted the maximial dispersal costs to 900 for random, 650 for clustered, and 400 for contiguous habitat allocation. In total we analysed 250 networks per network type random, clustered, and contiguous. See Fig 4(a)–4(d) for examples of the networks.

#### Standard networks

We created standard networks (random, regular, and small-world) using algorithms from the Python package NetworkX [47]. In random networks, two nodes are connected purely at random with uniform distribution and nodes usually have very similar degrees. They were generated using the algorithm proposed by [48]. Regular networks are networks, where every node has the same degree [37]. Small-world networks are a mixture of regular and random networks and represent the small-world phenomenon from the social sciences [37, 49]. While most nodes are not connected to each other, neighbours of a node are connected with particularly high probability. In other terms, small-world networks are highly clustered and at the same time also exhibit particularly low average shortest path distances. We used the algorithm proposed by [50] to construct small-world networks.

We created two sets of these standard networks varying in their number of links per network. For sparse standard networks, all parameters were set to create networks with a number of nodes and corresponding links similar to the landscape-based networks. This led to very sparse networks with only 4% of links present. Dense standard networks were also created with a number of nodes similar to the landscape-based networks, however the parameters were chosen such that about 75% of the potential links were present. S3.1 Table in S1 File shows the parameters and algorithms used to create the standard networks and Fig 4(e)–4(f) show examples of the networks.

In total, we analysed 250 networks per network type with the number of nodes between 50 and 111.

### Effect of used algorithms on the clustering coefficient

We evaluated the effect of the two proposed algorithms on the clustering coefficient and compared the results to randomly adding links. To this end *m* = 2 links were added to each of the created networks using (1) the Greedy algorithm, (2) the Lazy Greedy algorithm, and (3) a purely random approach. We compared these results with the clustering coefficient of the original network and the optimal solution, which was found by iterating over all pairs of potential links.

In this analysis, we considered both standard and landscape-based networks, as the heuristics to maximize the clustering coefficient can be applied to any network. We defined the set of potential links to be the set of all unconnected pairs of nodes E=V×V\E.

### Effect of used algorithms on robustness of habitat networks

In a last step, we evaluated how much the added links improved the robustness of landscape-based habitat networks against habitat loss. We applied the simulations introduced by Heer et al. [51] to simulate habitat loss and evaluate the habitat network’s robustness. For the simulations, a random habitat loss scenario was assumed where habitat patches (i.e., nodes) and corresponding links get lost permanently purely at random. On the remaining networks, random local extinctions were simulated, in a way that depends on the local-extinction risk of species and each patch’s neighbourhood. Empty habitat patches could then be recolonised through dispersal from connected colonised habitat patches, in a way that depends on the dispersal range of species and each patch’s neighbourhood. These extinction and recolonization processes were continued until a stationary distribution was reached. From this we obtain the fraction of colonised habitat patches. These simulations of habitat loss and subsequent extinction and recolonization processes were repeated for different degrees of habitat loss to obtain a robustness curve describing the fraction of colonised habitat patches in dependence on the fraction of lost habitat patches. Based on this robustness curve, we used the ‘area under the curve’ (AUC) as a measure to quantify metapopulation robustness. See S1 File and [51] for more details on the robustness simulation.

We compared the heuristics Greedy and Lazy Greedy with randomly adding links to the network and added 5 to 30 links in increments of 5. Baseline of these simulations was the robustness of the original habitat networks and we compared the increase of robustness originating from adding links using the different algorithms.

As the robustness simulations were specifically designed to evaluate the robustness of metapopulations on habitat networks, we considered the landscape-based habitat networks in this section only. We restrict the set of potential links E to those unconnected pairs of patches that are at most 2500 m apart from each other:

$$E≔\{\left(u,v\right)\in V\times V\backslash E\;|\;{\text{dist}}_{\text{Eucl}}\left(u,v\right)<2500\;\text{m}\}.$$

This represents the real world assumption, previously used in [38], that our generic species can traverse at maximum 2500 m of open agricultural land with the dispersal distance reducing for areas with lower permeability such as urban area and forestry.

## Results & discussion

### Effect of used algorithms on the clustering coefficient

**Fig 5:**
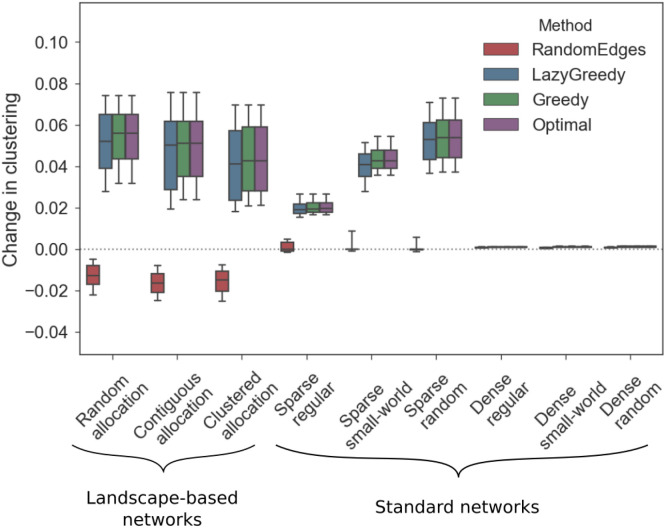
Greedy and Lazy Greedy algorithm applied to landscape-based and sparse networks lead to a higher increase in the clustering coefficient compared to randomly adding links. The horizontal axis shows the different network types, the vertical axis shows the change in clustering compared to the original network. The colour coding of the box-plots indicates the different algorithms.

To compare the different algorithms, we added two links to the networks using each of the algorithms and calculated the difference in the clustering coefficient between the extended network and the original one. The optimal solution of adding two links to the landscape-based networks increases the clustering coefficient by 0.05 on average. For the sparse networks, the optimal solution resulted in a mean increase between 0.02 (regular networks) to 0.04 (small-world networks). All three dense network types showed no increase in the clustering coefficient after two links were added (Fig 5).

Our proposed algorithms Lazy Greedy and Greedy return results close to the optimal solution with Lazy Greedy being slightly worse. For both the Greedy and optimal solution the mean increase in the clustering coefficient was 0.030 over all network types and for the Lazy Greedy solution the mean increase was 0.029.

Adding two links randomly decreases the clustering coefficient for almost all landscape-based networks with a mean decrease of 0.15. The clustering coefficient for standard networks (both sparse and dense) remains unchanged by adding two links randomly.

For sparse networks, this implies that applying our heuristics to identify new links has a much larger impact on the clustering coefficient compared to the random approach. The same holds for habitat networks, which are usually sparse, leading to the conclusion that both the Greedy and Lazy Greedy heuristic are preferable to randomly adding links to a habitat network. For dense networks, however, adding two links has almost no impact on the clustering coefficient, independent from the considered method. As the majority of nodes in dense networks has a particularly high degree, the impact of an additional link decreases (see Eq 1), which explains the different results for dense networks. Furthermore, the clustering coefficient of dense networks is already rather high, leading to a smaller potential increase as well.

**Fig 6:**
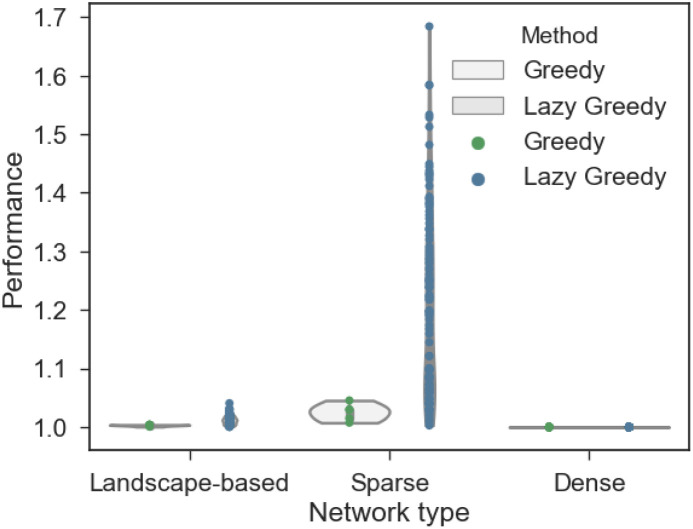
The Greedy algorithm returns an optimal solution in almost all cases. The vertical axis shows the quotient between optimal solution and solution of the heuristic. Only non optimal results are shown.

To quantify, how close the Greedy and Lazy Greedy algorithms approximate the optimal solution, we compared the clustering coefficient of the optimal solution with that produced by the Greedy and Lazy Greedy algorithm. The Greedy algorithm returned the optimal solution in 97.6% of the 2250 networks and the discrepancy between the clustering coefficient of the optimal solution and that produced by the Greedy algorithm was at most 3.8%. The Lazy Greedy algorithm, on the other hand, returned the optimal solution in only 76.0% of all networks and the discrepancy went up to 63.6%, increasing the clustering coefficient to 0.03 instead of 0.05 in that particular case (Fig 6).

### Effect of used algorithms on robustness of habitat networks

**Fig 7:**
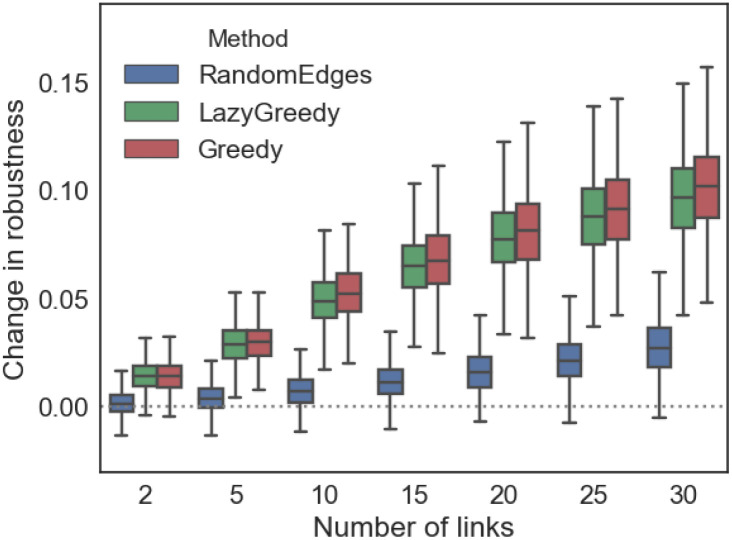
The robustness of networks increases with the number of additional links added using the Greedy or Lazy Greedy algorithm. The horizontal axis shows the number of links added to the network, the vertical axis the change in robustness. Colours indicate the algorithm used and each box shows the results over all landscape-based habitat networks.

The robustness of networks increased with the number of additional links, when the links were added with the help of the Greedy or Lazy Greedy algorithm. The correlation between the mean increase in robustness and number of additional links is *r* = 0.8 for the Greedy algorithm and *r* = 0.76 in case of the Lazy Greedy algorithm. If the links are added randomly, the increase in robustness is much smaller and the correlation between robustness and number of additional links drops to *r* = 0.54 (Fig 7).

These results strongly suggest that using the presented algorithms to identify the links that should be added to a habitat network results in a much higher increase in robustness compared to randomly adding links.

### Conclusion

We introduced two heuristics to maximise the clustering coefficient of a network by adding links. These methods work particularly well for sparse networks and yield a much higher increase in habitat network robustness compared to randomly adding links. Both the Greedy and Lazy Greedy heuristic return results close to the optimal solution for adding *m* = 2 links. While the Lazy Greedy algorithm is faster for large *m*, the Greedy algorithm returns results closer to the optimal solution and we suggest to apply the Greedy algorithm if possible.

Habitat connectivity is crucial for species survival, and habitat restoration efforts need to consider the robustness of habitat networks against habitat loss to increase connectivity and mitigate effects of future habitat loss. Our study shows that the location of links—and not only the number of links—has a large impact on metapopulation robustness, and presents a fast way to determine the best location for further links. It is the first study—to the best of our knowledge—that maximises the clustering coefficient of networks by adding links.

The heuristics presented here can be used to plan restoration efforts and increase habitat connectivity, as they provide locations in the habitat network that would lead to the largest increase of metapopulation robustness if they were connected. Simultaneously, our study shows that the location of links has a large impact on metapopulation robustness and thus emphasizes the importance of further mathematical models to improve habitat restoration strategies.

In summary, we presented two heuristics that identify which parts of a network need to be connected to obtain a higher network robustness. These heuristics work particularly well for habitat networks and increase metapopulation robustness with increasing number of links added.

## Supporting information

S1 File (PDF)
