# Supplementary material for: Maximising the clustering coefficient of networks and the effects on habitat network robustness
Source: PLoS One. 2020 Oct 20;15(10):e0240940. doi: 10.1371/journal.pone.0240940 (PMC7575089; doi:10.1371/journal.pone.0240940)
Supplement: S1 File — (PDF) [file pone.0240940.s001.pdf]

Maximising the clustering coefficient of networks and the effects on habitat network robustness

Supplementary Information

Henriette Heer[1,*], Lucas Streib[1], Ralf B. Schäfer[1], and Stefan Ruzika[2]

[1]Department of Quantitative Landscape Ecology, iES Landau, University of Koblenz-Landau, Fortstr. 7, 76829 Landau i.d. Pfalz, Germany
[2]Department of Mathematics, University of Kaiserslautern, 67653 Kaiserslautern, Germany
[*]Corresponding author, heer@uni-koblenz.de

1 Update clustering coefficient

We prove Eq 1:

Lemma 1.1. *Let $G = (V, E)$ be a network as above, $(u, v) \in (V \times V) \setminus E$ and $G' = (V, E \cup \{(u, v)\})$ be the network resulting from G by inserting (u, v). Then the following holds:*

1. $d'_u = d_u + 1$ *and* $d'_v = d_v + 1$
2. $T'(w) = T(w) + 1$ *for all common neighbours* $w \in N(u, v)$ *of* u *and* v
3. $T'(u) = T(u) + |N(u, v)|$ *and* $T'(v) = T(v) + |N(u, v)|$
4. $T'(w) = T(w)\ \forall w \in V \setminus N(u, v), w \neq u, v$
5. $d'_w = d_w$ *for all* $w \neq u, v$

Proof. Let G, e, G' as above.

1. Consider node u in G'. Then, v becomes a neighbour of u after (u, v) was inserted, i.e. v is a neighbour of u in G', but not in G. All other neighbours do not change. Thus $d'_u = d_u + 1$.
2. Consider $w \in N(u, v)$. Then uvw is a triangle in G', but not in G. It is the only triangle involving both w and (u, v). All other triangles involving w do not involve (u, v) and are therefore also in G and thus $T'(w) = T(w) + 1$.
3. Consider node $u \in V$. Triangles involving u in G' that are not in G also have to involve v. The missing node in a triangle thus has to be connected to both u and v. Thus exactly the neighbours of both u and v are involved in triangles in G' that do not exist in G.
4. Let $u, v \neq w \in V \setminus N(u, v)$. Triangles in G' that are not in G have to involve (u, v). Since w is not a neighbour of both u and v, no triangle uvw exists and thus $T'(w) = T(w)$.

□

We use Lemma 1.1 to calculate the clustering coefficient $C_{G'}$ of G' with help of C_G.

Lemma 1.2. *Let $G=(V,E)$ be a network as above with n nodes, $(u,v)\in\mathcal{E}$ and $G'=(V,E\cup\{(u,v)\})$. Let $k:=|N(u,v)|\geqslant 1$ be the number of common neighbours of u and v. Then the difference in clustering is as follows:*

$$\Delta C = C_{G'} - C_G = \frac{1}{n}\left(\Delta C(u) + \Delta C(v) + \sum_{w\in N(u,v)} \frac{2}{d_w(d_w-1)}\right) \tag{1}$$

with

$$\Delta C(u) = \begin{cases} \frac{2k(d_u-1)-4T(u)}{d_u(d_u^2-1)} & \text{if } d_u > 1 \\ 1 & \text{if } d_u = 1. \end{cases}$$

Proof. Let $u,v\in V$ with $d_u,d_v>1$ and set $k:=|N(u,v)|$ as the number of common neighbours. Then, it holds

$$\begin{aligned} C_{G'} &= \frac{1}{n}\left(C_{G'}(u)+C_{G'}(v)+\sum_{w\in N(u,v)} C_{G'}(w) + \sum_{w\notin N(u,v), w\neq u,v} C_{G'}(w)\right) \\ &= \frac{1}{n}\left(\frac{2T'(u)}{d'_u(d'_u-1)} + \frac{2T'(v)}{d'_v(d'_v-1)} + \sum_{w\in N(u,v)} \frac{2T'(w)}{d'_w(d'_w-1)} + \sum_{w\notin N(u,v), w\neq u,v} C_{G'}(w)\right) \\ &= \frac{1}{n}\left(\frac{2(T(u)+k)}{(d_u+1)d_u} + \frac{2(T(v)+k)}{(d_v+1)d_v} + \sum_{w\in N(u,v)} \frac{2(T(w)+1)}{d_w(d_w-1)} + \sum_{w\notin N(u,v), w\neq u,v} C_G(w)\right). \end{aligned}$$

The difference in clustering by inserting $e=(u,v)$ can be calculated as

$$\begin{aligned} n\Delta C =& C_{G'} - C_G \\ =& \frac{2(T(u)+k)}{(d_u+1)d_u} - \frac{2T(u)}{d_u(d_u-1)} + \frac{2(T(v)+k)}{(d_v+1)d_v} - \frac{2T(v)}{d_v(d_v-1)} + \sum_{w\in N(u,v)}\left(\frac{2(T(w)+1)}{d_w(d_w-1)} - \frac{2T(w)}{d_w(d_w-1)}\right) \\ &+ \sum_{w\notin N(u,v), w\neq u,v}\left(\frac{2T(w)}{d_w(d_w-1)} - \frac{2T(w)}{d_w(d_w-1)}\right) \\ =& \frac{2k(d_u-1)-4T(u)}{d_u(d_u^2-1)} + \frac{2k(d_v-1)-4T(v)}{d_v(d_v^2-1)} + \sum_{w\in N(u,v)} \frac{2}{d_w(d_w-1)}. \end{aligned}$$

Now, consider $u,v\in V$ with degree 1 and a common neighbour w. Then the difference in clustering

is:

$$\begin{aligned}
n\Delta C &= C_{G'} - C_G \\
&= \frac{2(T(u)+k)}{2} - C_G(u) + \frac{2(T(v)+k)}{2} - C_G(v) + \sum_{w\in N(u,v)} \frac{2}{d_w(d_w-1)} \\
&= 1 - 0 + 1 - 0 + \frac{2}{d_w(d_w-1)} \\
&= 2 + \frac{2}{d_w(d_w-1)}.
\end{aligned}$$

This equation holds, because $d_u = d_v = 1$ and thus $T(u)$, $T(v)$, $C(u)$, and $C(v)$ all equal zero and $k = 1$, as they have a common neighbour. □

2 Robustness simulation

We simulated habitat loss and subsequent metapopulation dynamics as proposed by Heer et al. (under review) on the landscape-based habitat networks to evaluate the increase of metapopulation robustness on those networks. Here, we briefly summarize how the simulation was modelled to make it easier for readers to follow our findings.

2.1 Simulation overview

To evaluate the robustness of a habitat network against habitat loss, we first simulated the habitat loss by randomly removing habitat patches from the network. Habitat patches on the remaining network were assumed to be fully colonised. Then, metapopulation dynamics consisting of local extinctions and subsequent recolonisation from neighbouring patches were simulated until a stationary distribution was reached. This process of simulated habitat loss and subsequent metapopulation dynamics was then repeated for different degrees of habitat loss to obtain a robustness curve describing the fraction of colonised habitat patches in dependence on the fraction of lost habitat patches. Based on this robustness curve, we used the 'area under the curve' (AUC) as a measure to quantify metapopulation robustness: the higher the fraction of colonised habitat patches across fractions of lost habitat patches, the higher the AUC, and thus the estimated metapopulation robustness. For each network, simulations were replicated ten times to average over the sources of randomness affecting habitat loss and metapopulation dynamics.

2.2 Habitat loss

We assumed a random habitat loss scenario, which removed each habitat patch with equal probability p.

2.3 Metapopulation dynamics

For a given level of habitat loss, metapopulation dynamics were simulated on the remaining habitat network, by considering local extinctions in habitat patches and the recolonization of habitat patches. We used the size of cliques to measure, how well a patch is connected within its neighbourhood, as the survival of a population in a habitat patch depends on its potential to exchange individuals with neighbouring patches. Denoting by $c(v)$ the size of the largest clique that contains the node v we assumed that the population in v goes extinct with probability

$$p_{\text{ext}}(v) = a^{1-c(v)},$$

where $a > 1$ is a species-specific parameter governing the local-extinction risk of a species. We can think of these risks decreasing with increasing $c(v)$ more slowly for habitat specialists (small values of a) and more rapidly for habitat generalists (large values of a). We investigated species with three different levels of local-extinction risks - low ($a = 2$), medium ($a = 5$), and high ($a = 9$).

Empty habitat patches can be recolonised from connected colonised patches. Recolonisation was modelled with the help of a Gaussian dispersal kernel and we assumed that an empty habitat patch v becomes recolonised from a colonised patch w with probability

$$p_{\text{col}}(v, w) = \frac{m_{vw}}{\sum_{u \in V} m_{uw}},$$

where $m_{vw} = \exp(-\frac{1}{2} d_{vw}^2 / \sigma^2)$ is the dispersal kernel, V the set of all network nodes, d_{vw} the distance between habitat patches v and w in terms of dispersal costs and $\sigma > 0$ a species-specific dispersal parameter governing the dispersal range of a species. We can think of these dispersal ranges as being low for poor dispersers (small values of σ) and high for good dispersers (large values for σ). Similar to a, we investigate values of $\sigma \in [2, 5, 9]$ to account for the different dispersal capacities of different species.

These local extinctions in and recolonizations of habitat patches were simulated alternately until a stationary frequency of colonized patches was reached.

3 Networks

Network	NetworkX algorithm	Parameter sparse	Parameter dense
Regular	nx.random_regular_graph	$d = 4$	$d = 58$
Random	nx.erdos_renyi_graph	$p = 0.04$	$p = 0.75$
Small-world	nx.newman_watts_strogatz_graph	$k = 2$, $p = 0.6$	$k = 39$, $p = 0.5$

Table 1: **Parameters to create standard networks.** d is the degree of each node, p denotes the percentage of links present in the network and k is the degree of each node in the small-world network before rewiring.
